# Prenatal Stress: Molecular Mechanisms and Cardiovascular Disease

**DOI:** 10.5935/abc.20180246

**Published:** 2019-01

**Authors:** Marcelo Diarcadia Mariano Cezar, Mariana Janini Gomes, Ricardo Luiz Damatto

**Affiliations:** 1 Faculdade de Ciências Sociais e Agrárias de Itapeva (FAIT), Itapeva, SP - Brazil; 2 Faculdade de Medicina de Botucatu - Universidade Estadual Paulista (UNESP), Botucatu, SP - Brasil

**Keywords:** Stress, Psychological, Cardiovascular Diseases, Hypertension, Heart Failure, Rats

Concern of the researchers in finding the causes of cardiovascular disorders is evident. Recently, scientists are focusing in intrauterine environment investigations in order to seek early causes of these diseases.^1^ Studies indicates that prenatal stress increases risk of cardiovascular diseases in adulthood.^2^^,^^3^ Among the risks, susceptibility to adult hypertension is a concern, and the sympathetic nervous system is one of the targets of interest, specifically the activity of beta-adrenergic receptors, which has subtype β1 cardiac predominance.^4^^,^^5^ These receptors modulates cardiac changes and may lead to ventricular dysfunction as well as severe conditions of heart failure,^6^ which increases mortality risk as showed in hypertensive rats studies.^7^^,^^8^

Another factor associated with the occurrence of heart failure is monoamine oxidase A (MAO-A). This enzyme has its activity increased in hypertension and is responsible for the degradation of catecholamine, which increases the reactive oxygen species generation leading to cardiotoxicity.^9^ Therefore, it is extremely important target the causes to prevent or attenuate the of alterations resulting from cardiovascular diseases.

Jevjdovic et al.^10^ developed a study in order to investigate region-specific gene expression of adrenergic receptors subtypes (ADRB1, ADRB2 e ADRB3) and of the MAO-A in the female and male offspring myocardium. The mentioned study is one of the few studies that evaluated the difference between gender and regions of left ventricle (apex and base).

The authors highlighted that the occurrence of a stressful situation increased the plasmatic level of the adrenocorticotrophic hormone (ACTH), which characterizes maternal stress. However, prenatal stress did not cause changes in the gestational period on the evaluated parameters, such as maternal weight gain, water and food consumption, blood glucose, litter size, neonatal weight and offspring weight gain, with the latter as one of the main risk factors for the development of cardiovascular diseases in adults.^10^

Adrenergic receptors evaluation has elucidated a decrease of ADRB1 expression in the left ventricle apical region in female offspring. The reduction of ADRB1 apical expression is characteristic of cardiac diseases. ADRB3 expression was undetectable in rats left ventricle. In addition, the authors did not found significant modifications in mRNA level of MAO-A in female or male prenatal heart. This was the very first study that reported gene expression level of the β adrenergic receptor in different regions of rats left ventricle.^10^

Jevjdovic et al.^10^ showed very relevant data regarding sex-related effects of prenatal stress on region-specific expression in heart rats. The results of gene expression suggests that prenatal stress may lead to cardiovascular diseases. However, protein expression evaluation would be important to corroborate and consolidate the statements of the mentioned article.
